# Therapeutic Dilemmas Arising From a Serpentine Thrombus Across a Patent Foramen Ovale in a Patient With Dilated Cardiomyopathy

**DOI:** 10.7759/cureus.75346

**Published:** 2024-12-08

**Authors:** Garvit Nama, Mohammad Khurram Nadeem

**Affiliations:** 1 Internal Medicine, Luton and Dunstable University Hospital, Luton, GBR; 2 Medicine, University Hospitals Bristol and Weston NHS Foundation Trust, Bristol, GBR; 3 Cardiology/Internal Medicine, Luton and Dunstable University Hospital, Luton, GBR

**Keywords:** dilated cardiomyopthy, mri cardiac, patent foramen ovale (pfo), pulmonary embolism (pe), transthoracic echocardiogram

## Abstract

A thrombus straddling a patent foramen ovale (TSPFO) is a rare condition that presents significant health risks, including stroke or myocardial infarction, and can be life-threatening if not promptly addressed. We report the case of a 42-year-old female with morbid obesity who presented with sudden shortness of breath due to a bilateral pulmonary embolism. Imaging revealed a thrombus extending from the right atrium to the left atrium through the patent foramen ovale (PFO). Further evaluation showed a new diagnosis of dilated cardiomyopathy, resulting in biventricular heart failure, confirmed by cardiac MRI. Given her relatively stable hemodynamics during her hospital stay, the patient was successfully treated with anticoagulation, leading to complete resolution of the clot, thus avoiding the need for thrombolysis or surgical intervention. In managing TSPFO, treatment decisions should be based on factors such as hemodynamic stability, coexisting conditions, the acute nature of the diagnosis, and the risk of embolism. While some literature reviews and case studies suggest that surgery may lower the risk of complications compared to thrombolysis or conservative medical management, the lack of level I and II evidence highlights the need for an individualized, evidence-informed approach to effective risk management.

## Introduction

A thrombus straddling a patent foramen ovale (TSPFO) is a rare and seldom-reported phenomenon in which a clot passes from the right atrium to the left atrium through a patent foramen ovale (PFO). This condition carries a very high risk of myocardial infarction (MI) and ischemic stroke (IS) [1]. While dilated cardiomyopathy (DCM) is known to increase the risk of intracardiac thrombus formation due to reduced ejection fraction and chamber dilation, which leads to blood stasis and a hypercoagulable state [2,3], there is limited literature on the coexistence of DCM and a thrombus crossing a PFO. In this report, we present the case of a patient who developed a pulmonary embolism (PE) and a new diagnosis of DCM, with a transthoracic echocardiogram (TTE) capturing a thrombus straddling the PFO.

## Case presentation

A 42-year-old female with morbid obesity (BMI of 41) sought urgent care in the emergency department, reporting an abrupt onset of nocturnal dyspnea and tachypnea, along with a one-week history of bilateral lower extremity edema. The event had woken her from sleep and was associated with non-radiating, pleuritic chest pain, rated 8/10 on the pain scale, and a sharp sensation in both costal angles when taking deep breaths over the past four to five hours. The bilateral lower extremity edema was new, symmetrical, extending to the hips, pitting, and had slowly worsened over the last seven days. There was no history of fever, central or heavy chest pain, syncope, palpitations, or dizziness. Notably, she had experienced a provoked PE during her first pregnancy 19 years ago, which had been treated with three months of low molecular weight heparin therapy. She was not taking any contraceptives and denied any recent significant immobilization. There was no relevant family history.

Upon initial assessment, she was tachycardic at 109 beats per minute, hypoxic (85% on room air), tachypneic at 40 breaths per minute, hypertensive (160/91 mmHg), and afebrile, indicating clinical shock due to respiratory failure. Her PE Wells score was 6 (PE likely diagnosis, previous PE, and tachypnea). Chest auscultation revealed bibasal coarse crepitations, while cardiovascular examination showed elevated jugular venous pressure, normal heart sounds, and bilateral, symmetrical pitting edema extending to the hips. Neurological examination was normal, and there were no clinical signs suggestive of deep vein thrombosis (DVT), such as unilateral calf tenderness, erythema, or edema. The patient also reported mild vaginal bleeding, consistent with her menstrual period.

**Figure 1:**
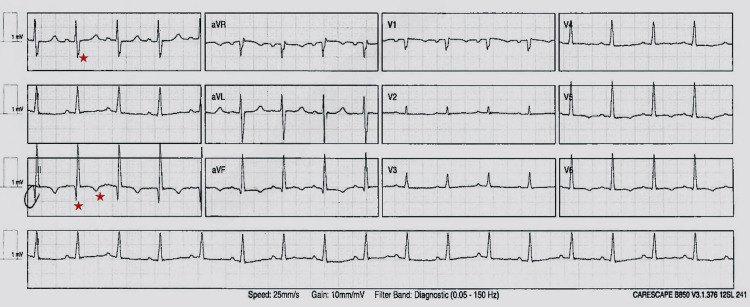
ECG showing the S1Q3T3 pattern (highlighted with a red star), characterized by a deep S wave in lead I, a deep Q wave, and an inverted T wave in lead III, commonly seen in PE PE, pulmonary embolism

Her blood gas indicated type 1 respiratory failure with normal pH (pO2 9.7 kPa, pCO2 5 kPa, pH 7.42, and bicarbonate 25 mmol/L). The ECG showed the classic S1Q3T3 pattern (although neither sensitive nor specific for the diagnosis) with sinus tachycardia and no axis deviation or bundle branch block (Figure 1). Chest X-ray revealed cardiomegaly with central vascular enlargement indicative of cardiac failure, with no obvious pleural effusion or pneumothorax. Blood biochemistry showed a raised age-adjusted D-dimer of 2616 ng/ml, high creatinine of 151 micromol/L, CRP of 45 mg/L, hemoglobin of 95 gm/L (chronic microcytic anemia), white cell count of 11,000/µL, cardiac troponin T of 40 ng/L, N-terminal pro-B-type natriuretic peptide levels of 3,703 pg/ml, and normal liver function and coagulation screen (Table 1).

**Table 1:** Lab values with reference range NT-ProBNP, N-terminal pro-B-type natriuretic peptide

Labs	Value	Reference range
pH	7.42	7.35-7.45
pO2	9.7 kPa	11-14 kPa
pCO2	5 kPa	4-6 kPa
Bicarbonate	25 mmol/L	22-28 mmol/L
D-dimer	2,616 ng/ml	<500 ng/ml
Creatinine	151 micromol/L	60-100 micromol/L
CRP	45 mg/L	<5 mg/L
Hemoglobin	95 gm/L	120-150 gm/L
White cell count	11,000/microliter	4,000-11,000/microliter
Cardiac troponin T	40 ng/L	<11 ng/L
NT-ProBNP	3,703 pg/ml	<400 pg/ml

**Figure 2:**
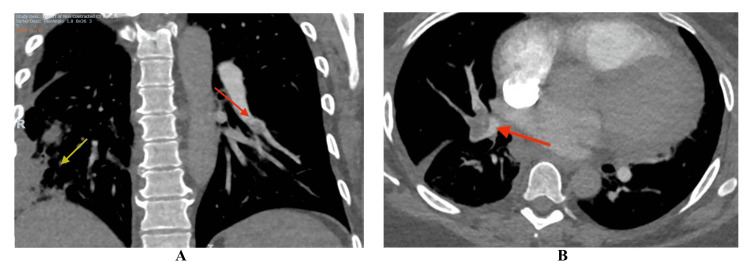
CTPA (pulmonary arterial phase, 10 seconds post-contrast injection) showing filling defects (red arrow) in the right and left interlobar arteries, along with right lung infarcts (yellow arrow) (A) CTPA (coronal section) showing focal areas of heterogeneous, wedge-shaped consolidation in the right lower lobe (yellow arrow) and filling defects in the left segmental pulmonary arteries (red arrow). (B) CTPA (transverse section) showing filling defects in the right segmental pulmonary arteries (red arrow). CTPA, CT of the pulmonary arteries

Her CT of pulmonary arteries (CTPA) revealed filling defects in the bilateral segmental and subsegmental arteries, along with evidence of contrast reflux into the inferior vena cava and interventricular septal flattening, indicating right heart strain and a high clot burden. Additionally, focal areas of heterogeneous, wedge-shaped consolidation were observed in the right lung, consistent with lung infarcts (Figure 2).

**Figure 3:**
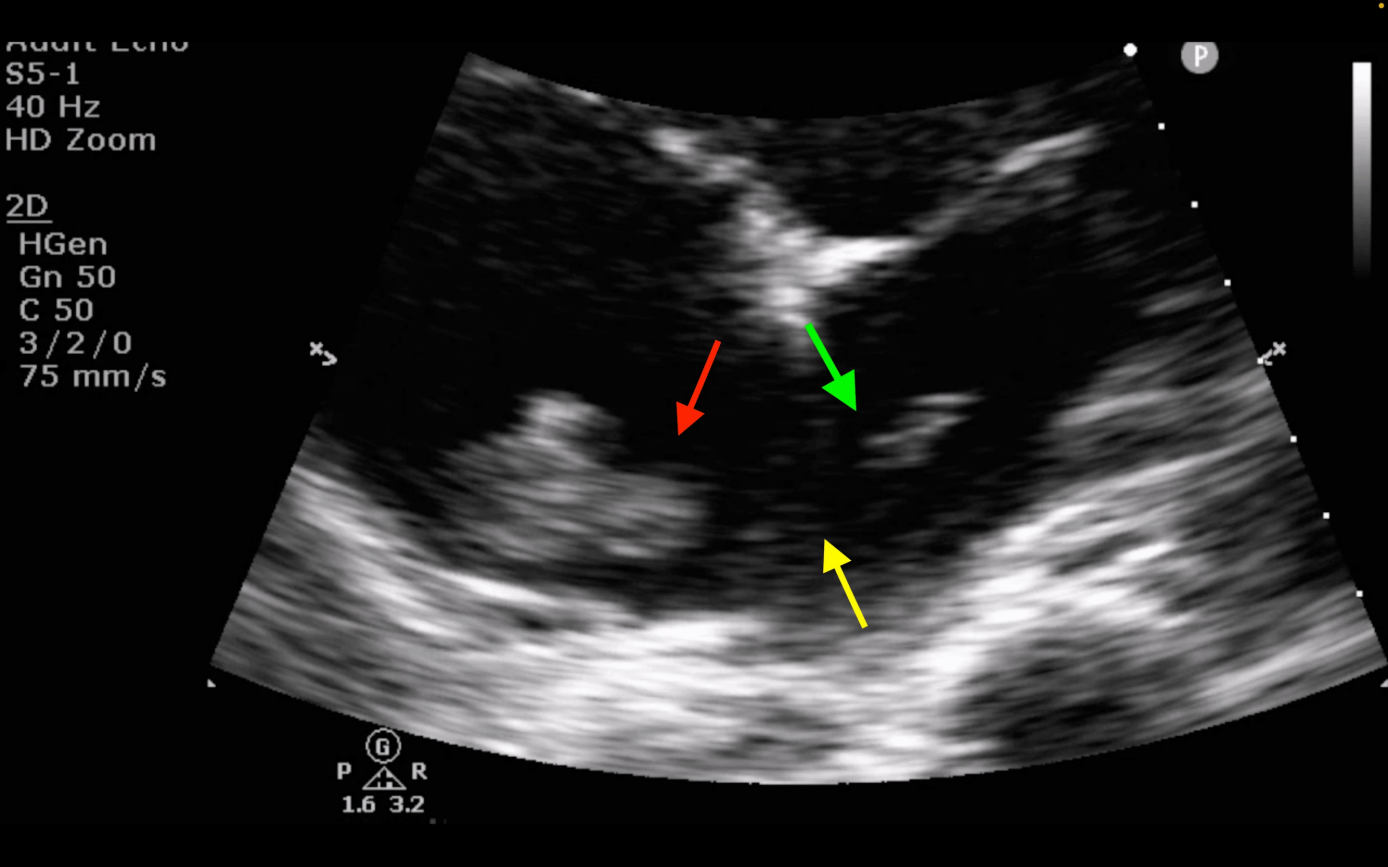
TTE showing a hyperechoic mass in the right atrium (red arrow) passing through the interatrial septum (yellow arrow) and appearing in the left atrium (green arrow), suggestive of a TSPFO TSPFO, thrombus straddling a patent foramen ovale; TTE, transthoracic echocardiogram

TTE revealed a serpentine thrombus straddling the PFO, along with a markedly reduced left ventricular ejection fraction (LVEF) of 20-25% based on visual estimation, global hypokinesia, and high pulmonary artery systolic pressure (PASP) of 84 mmHg. Moderate tricuspid regurgitation was observed, indicating a high clot burden and raised pulmonary artery pressures, which contributed to signs of right-sided heart failure, such as bilateral extremity edema (Video 1, Figure 3). The right ventricle was dilated with reduced radial and longitudinal function. Additionally, a small, localized pericardial effusion adjacent to the right atrium was noted, although it had no obvious hemodynamic significance. Her international normalized ratio, prothrombin time, and partial thromboplastin time were within normal limits. Thrombophilia and anti-phospholipid antibody screening were negative, and lupus anticoagulant was weakly positive, which, in the context of acute anticoagulation therapy, was interpreted as a false positive. Ultrasound imaging of the proximal deep veins in the lower limbs did not show any thrombus, although image quality was compromised due to peripheral edema, which hindered visualization of the deep veins. A CT scan of the chest, abdomen, and pelvis did not reveal any radiological evidence of malignancy.

**Video 1:** TTE showing a serpentine mass oscillating in the right atrium and crossing into the left atrium through a PFO PFO, patent foramen ovale; TTE, transthoracic echocardiogram

Following the initial presentation, the patient received anticoagulation as per the Pulmonary Embolism Response Team, beginning with subcutaneous heparin and later transitioning to an IV heparin infusion at 2,000 IU/hr due to pre-renal acute kidney injury (AKI stage 1, with creatinine rising to 1.7 times the baseline). A slow infusion of furosemide was initiated to manage peripheral edema, with continuous monitoring of blood pressure and urine output. Collaborative consultations with regional interventional cardiology, interventional radiology, and cardiothoracic surgery specialists led to the decision to pursue medical management, with anticoagulation and potential thrombolysis should the patient experience hemodynamic instability or arterial embolism. Due to her high BMI and severe biventricular systolic dysfunction, she was classified as a high-risk candidate for surgical intervention.

The patient was transferred to the ICU due to significant oxygen requirements from a large PE burden, with a Pulmonary Embolism Severity Index (PESI) of 111 (high risk range: 106-125). During her ICU stay, she developed a UTI, which was treated with an IV co-amoxiclav for five days. Her AKI improved with supportive measures, eliminating the need for hemofiltration. A follow-up TTE on day 6 showed a persistent severe reduction in the ejection fraction of both ventricles, but with a reduction in the size of the thrombus across the PFO. Additionally, her PASP decreased to 56 mmHg. The patient did not experience any hemodynamic compromise, MI, or IS. As her symptoms improved during the ICU stay, she was safely stepped down to a respiratory ward.

Further discussions with the regional pulmonary hypertension team recommended reconsideration of surgical or percutaneous thrombectomy, due to the continued risk of paradoxical embolism. On day 23 of admission, the patient was transferred to the regional cardiology center, where repeat imaging (TTE, CTPA, and cardiac magnetic resonance MRI) showed no evidence of intracardiac thrombus. CT of the coronary arteries revealed a calcium score of 0, no plaques, and a normal aorta. Cardiac MRI showed severe biventricular dilatation with reduced systolic function (LVEF 16%, right ventricular ejection fraction 19%), but no late gadolinium enhancement indicative of scar or infarct (Video 2). These findings were consistent with DCM, and she was referred to a heart failure clinic. The patient was discharged on day 27 of her hospital admission.

**Video 2:** Cardiac MRI cine, four-chamber view, showing severely dilated left and right ventricles, with no evidence of an intracardiac thrombus

The patient presented to the hospital after a two-month interval, manifesting acute decompensation of congestive cardiac failure. IV diuretics were started and transitioned to oral tablets following sufficient diuresis. The patient remained on direct-acting oral anticoagulants and guideline-directed medical therapy (GDMT) for heart failure and was compliant with her medications. At the four-month follow-up in the heart failure clinic, the patient reported notable improvement, with no apparent signs of fluid overload and a functional status of NYHA class II.

In accordance with the European Society of Cardiology (ESC) guidelines [4], given the high risk of left-sided thromboembolism, prophylactic closure of the PFO was recommended in an outpatient follow-up with the interventional cardiology clinic. However, the patient opted against the procedure, weighing the risks and benefits, and expressed confidence in the protective efficacy of oral anticoagulants in preventing IS and MI. After the initial event, the patient experienced no further admissions due to decompensated heart failure and maintained symptomatic stability at the 12-month heart failure clinic follow-up. In alignment with the ESC cardiomyopathy guidelines [5], the patient was offered genetic testing, which revealed no genetic cause for her cardiomyopathy.

## Discussion

The prevalence of a PFO in the general population ranges from 20% to 34% [6], although the occurrence of a live thrombus-in-transit crossing through the PFO is exceedingly rare, with fewer than 400 documented cases since Nellessen's initial report in 1985 [1]. This condition significantly increases the risk of paradoxical embolism, where a right-sided thrombus crosses the PFO and leads to MI or IS, necessitating prompt intervention. As highlighted by Shah et al. in their comprehensive statistical analysis of TSPFO in 2021 [7], patients typically present with symptoms such as dyspnea, chest pain, and syncope, with PE or DVT often identified as the underlying cause.

DCM further contributes to the formation of intracardiac thrombi [3,8,9], increasing the risk of embolic phenomena. Timely identification of a thrombus-in-transit is crucial, particularly in at-risk patients presenting with embolism in the arterial circulation [8]. Evaluating the risk of thrombus embolization, involving active patient participation, and collaborative decision-making in interdisciplinary teams - including cardiology, interventional radiology, and cardiothoracic surgery - are essential for effective management [7,10-12].

Baydoun et al. proposed a stepwise approach in 2013, categorizing patients based on their hemodynamic status and selecting the appropriate treatment strategy. In our case, given the patient’s high surgical risk, anticoagulation therapy alone was chosen as the appropriate management [13]. However, due to the exceptional rarity of this event and the absence of Level I and II evidence, the ideal treatment approach remains unclear. Current systematic reviews favor surgical management as the preferred strategy [7,10,11,14], showing comparable mortality rates to medical management but significantly reducing the risk of MI and IS. Thrombolysis, although associated with high mortality rates, is generally reserved for the most unstable patients [13,14].

In this case, TSPFO was successfully resolved with anticoagulation therapy, and no paradoxical embolism occurred. Therefore, it is critical to balance the risks, urgency, and benefits of surgical and medical management while involving patients in the decision-making process to achieve optimal outcomes.

## Conclusions

We present a rare case of a TSPFO, highlighting several important considerations in the management of PE and its related complications. In evaluating PE, it is recommended to use objective scoring systems, such as the PE Wells score to guide investigations, and the PESI for risk stratification regarding 30-day mortality. For diagnosing a thrombus across the PFO, initial evaluation should include transthoracic echocardiography, with escalation to transesophageal echocardiography when further characterization is needed. The optimal treatment strategy for a thrombus across a PFO remains uncertain; therefore, an interdisciplinary approach is essential, with active patient engagement in the decision-making process to tailor individualized treatment plans. Additionally, managing DCM requires careful titration of GDMT, along with family screening and genetic risk assessment to evaluate the risk of sudden cardiac death. Overall, these findings underscore the importance of a comprehensive and collaborative approach to managing patients with thromboembolic phenomena in the context of a PFO and compromised cardiac function.
